# Supplementary material for: Multiple Case Studies in German Children with Dyslexia: Characterization of Phonological, Auditory, Visual, and Cerebellar Processing on the Group and Individual Levels
Source: Brain Sci. 2022 Sep 25;12(10):1292. doi: 10.3390/brainsci12101292 (PMC9599942; doi:10.3390/brainsci12101292)
Supplement: Supplementary file 1 [file brainsci-12-01292-s001.zip › brainsci-1892803-supplementary.pdf]

S1. Standardized Tests

S1.1 IQ

As a measure of nonverbal intelligence, the Performance IQ of HAWIK-III [55] was applied. The HAWIK-III is the German adaption of the third edition of the American Wechsler Intelligence Scale for Children (WISC-III). Wechsler's definition of intelligence is described as an individual's ability to adapt and constructively solve problems in the environment. The Wechsler scales do not measure the quantity of intelligence, but instead measures one's intellectual performance. Performance (nonverbal) IQ of HAWIK-III is based on several subtests: Picture Completion (Bilder ergänzen, BE), Coding (Zahlen-Symbol-Test, ZS), Picture Arrangement (Bilder ordnen, BO), Block Design (Mosaiktest, MT), Object Assembly (Figurenlegen, FL). The performance subtests are nonverbal problems, all of which are timed and some of which allow bonus points for extra fast work. In Picture Completion subjects have to identify missing parts of pictures. In Coding subjects have to transcribe a digit-symbol code as quickly as possible for two minutes (eight and older). In Picture Arrangement subjects have to sequence cartoon pictures to make sensible stories. In Block Design subjects have to copy small geometric designs with four or nine larger plastic cubes. In Object Assembly subjects have to puzzle cut-apart silhouette objects with no outline pieces.

S1.2 Reading and Spelling

Reading fluency and accuracy both in single word as well as in text reading was assessed by means of a standardized reading test (Zürcher Lesetes, ZLT, [56]). In this test subjects read different lists of single words as well as different texts respective to their class level. The test acquires scores for the time needed as well as the errors made while reading the target words and texts.

Spelling performance was assessed by dictation of a gap text by means of standardized spelling tests appropriate for the respective class level: Westermann Rechtschreibtest 6+ (WRT 6+ [57]); Diagnostischer Rechtschreibtest für 4. Klassen (DRT 4 [58]), and Diagnostischer Rechtschreibtest für 5. Klassen (DRT5 [59]). The test acquires the number of wrongly spelled words.

S1.3 Handedness

Handedness was assessed by the Hand-Dominanz-Test (HDT,[54]). The HDT allows statements about the degree of handedness. It consists of three subtests: *Tracing*, *Dotting Circles* and *Dotting Squares*. Each subtest must be processed once with the left hand and once with the right hand. The performance superiority of a hand is determined computationally, and the hand dominance score can theoretically vary from -100 (extreme left-handedness) to +100 (extreme right-handedness).

S1.4 ADHD

One of the exclusion criteria was the coexistence of ADHD. ADHD is characterized by a trias of symptoms (inattention, impulsivity and hyperactivity). Inattention and impulsivity are the two cognitive domains leading to the most negative impact on cognitive performance in ADHD children. We thus chose three subtests of the Testbatterie zur Aufmerksamkeitsprüfung für Kinder (KiTAP, [53]) in order to address the domains inattention and impulsivity: *GoNogo* (as a measure for inhibition ability and impulse control), *Distractability* (a measure of willful control of

the focus of attention) as well as *Sustained Attention* (measure of longer-term maintenance of attention with high target stimulus density). In all three subtests performance parameters were acquired for total number of correct answers, total number of omission errors (missed responses on the target stimuli as a sign of inattentive working style), total number of commission errors (unrequired responses as a sign for impulsive working style) as well as reaction time (median of reaction time for all stimuli with a button press of the respective subtest).

One aspect of *Attention*, beside the control processes by which we take in information from the environment, is also the control of our reactions and behavior. This includes both the decision whether and how to react and the continuous control of our actions. One of the fundamental processes here is impulse control, i.e., the ability to suppress an inappropriate response. The simplest test for detecting impulsive response tendencies is the so-called *GoNogo* tasks.

Within the *GoNogo* subtest the children either saw the picture of a bat (target *Go*-stimulus) or a cat (irrelevant *NoGo*-stimulus). The task was to press a button on a computer mouse on the appearance of the bat.

One of the fundamental aspects of focused attention is the ability to willfully keep the focus under control even in complex situations and under distracting conditions. Children with ADHD for example are characterized by their high level of distractibility, as a result they often forget their goals from one moment to the next when another aspect catches their attention. A low level of distractibility is therefore an important prerequisite for concentrated work and is likely to be of great importance for schoolchildren in particular.

In the test *Distractability*, children worked on a GoNogo paradigm. A sad (target Go-stimulus requiring a button press) or a happy ghost (NoGo-stimulus, no button press) appeared within the castle's door. At the same time, strikingly colorful pictures appeared in the periphery during half of the trials, which should not be focused on visually. The difference between Go and Nogo stimuli can only be detected when the child stays visually focused. Therefore, changing the direction of gaze by paying attention to the distracting stimulus will result in missed critical stimuli and/or incorrect responses.

In the task *Sustained Attention*, continuous attention over a longer period of time (*concentration*) is tested. In comparison to vigilance, which is understood as the maintenance of attention under monotonous, low-stimulus conditions, the requirements of sustained attention are more complex. The conditions of sustained attention thus come closer to the demands of everyday life than pure vigilance tasks. In the test *Sustained Attention*, one stimulus must be compared with the following stimulus in order to decide whether they match in a given stimulus feature. The subject compared the stimuli regarding whether the ghosts appearing one after the other at different windows of a castle were of different colours. Subjects had to press a button if two ghosts of the same color appeared one after the other (*target stimulus*).

S2. Behavioral Testbattery

Table S1: Components of the behavioral test battery

Deficit Theory	Phonology			
	Phonological awareness	*Phonological recoding in the working memory*	*Phonological recoding with access of the semantic lexicon*	*Pseudoword Reading*
Tasks	segmentation of pseudowords	repetition of digit sequences	rapid naming of black and white objects	One-syllable pseudowords
	vowel substitution		rapid naming of coloured objects	Three-syllable pseudowords
	Swaping of phonemes		rapid naming of lists of letters.	
	Judging similarity of speech sound categories			
	Judging similarity of speech sounds			
	reverse speech sounds of a word			

Deficit Theory	Auditory	Visual			Cerebellar
		parvocellular	*magnocellular*	*other*	
Tasks	single tones	pattern sequence	coherent motion	rotation tasks	tapping
		coherent color	moving gratings		
		stationary/non-moving gratings			
	tone sequence syllables				statue

S2.1 Phonological Performance

Intensive research from the 1970s and 1980s identified three areas of phonological processing that play a major role in learning to read: phonological awareness, recoding in phonological working memory, and access to the lexicon. Wagner and Torgesen [25] have presented this very well in a review, including the multiple roles that phonological processing can play in learning to read (prerequisite for reading acquisition, facilitating element in reading acquisition, and consequence of reading acquisition). To assess these three phonological areas, we used various tests.

Phonological awareness

Phonological awareness describes the ability to analyze and operate with the sound structure of the spoken language and is supposed to be a major key factor for successful development of reading and writing skills. The tests Test zur Bestimmung der phonologischen Bewusstheit bei ältern

Schulkindern (PTB, [60]) and the test Basiskompetenzen für Lese-Rechtschreibleistungen (BAKO, [61]), assess the phonological awareness "in an narrower sense" as defined by Skowronek & Marx [62] in children in elementary school. The BAKO and PTB have 5 subtests in common: segmentation/disassembly of a pseudoword in its speech sounds (phonemes) (Pseudowortsegmentierung, which sounds do you hear in skop: s-k-o-p) (2) vowel substitution (Vokalersetzung, substitution of a certain vowel (a) through another vowel (i): the pseudoword "flap" becomes "flip"). (3) Swaping of speech sounds (Phonemvertauschung, swaping the first two speech sounds of a word: umka becomes muka) (4) Judging similarity of speech sounds (Lautkategorisierung, judging a row of words according to similar speech sound at the end: which word does not fit: haum-laum-faun-gaum) (5) reverse speech sounds of a word (Wortumkehr: sounding out a word reversedly: omel-lemo).

Phonological recoding in the working memory system

The ability to keep the representation of acoustic sound information of written symbols „alive" in short term memory is very crucial for the beginning reader. The beginning readers reads words sound by sound. The memory system has to be able to keep up this sound information in the short-term memory, in order to assemble the complete sound structure of the word and in order to access the meaning of the word.

The capacity of the phonological working memory was tested by the repetition of number sequences using a subtest of the HAWIK-III. The tasks mentioned already above for swapping of speech sounds as well as reversion of speech sounds also assess the phonological memory capacity.

Phonological recoding with access of the semantic lexicon

A fast and automatized recall of learned / knowledge based elements within the long-term memory is also an important ability needed for a fast and efficient reading process. In order to address the knowledge base in the long-term memory system, a written symbol (word or picture) has to be recoded. For this recoding, the phonological code within the long-term memory has to be accessed sufficiently fast in order to recall elements of the long-term memory.

This memory access was tested by the rapid naming of objects (BISC, [63]) as well as the rapid naming of lists of letters. In rapid naming, the children first rehearsed: a series of colored drawings of vegetables and fruits (tomato/red, salad/green, lemon/yellow, plum/blue) is shown to the child. The child is presented a list of colored and uncolored objects and is asked to name the colors of the objects. Afterwards, the child is presented a list of uncolored objects and is asked to name the according colors as fast as possible. As a third condition, the child is presented a list of objects which are colored in the wrong way (prune/red, tomato/green, lemon/blue, lettuce/yellow). The child is asked to name the correct colors of the objects as fast as possible.

In rapid letter naming the children had to name the names of a list of different lower and uppercase letters.

Pseudoword reading

Two reading tasks (non-standardized lists of pseudowords and regular words) were additionally administered, in order to operationalize different aspects of the reading process .

Pseudowords were pronounceable words according to the German language. Due to the fact that these pseudowords do not contain semantic information, they cannot be read via direct lexical access. Instead these pseudowords can only be read via grapheme-phoneme-conversion, thus through a process using assembled phonological strategies. The list

contained 15 pseudoword with 3-syllables and 15 pseudowords with 3 syllables. Pseudowords were constructed by conversion of vowels and consonants of real 3-sylallble words.

Reading of frequently used words is accomplished by the experienced reader through direct lexical access of lexical entries and thus is called addressed phonological reading. The list contained 15 words with 1 syllable and 15 words with 3 syllables. The word material was derived from the basic vocabulary adequate for the 5th to 9th grade of German students. User frequency of the word material was medium to high frequency range.

S2.2 Auditory Performance

We used three different auditory paradigms in order to investigate auditory sensory processing at different levels, from basic sensory auditory processing of single tones and tone sequences without linguistic reference or content to auditory syllable processing (with linguistic reference).

Attentive Tone Task: Pitch Differentiation

The task *Pitch Differentiation* addresses auditory processing on the simplest level. The Children heard two different tones and had to decide via button press whether they heard a high (1050 Hz, press right button) or low tone (1000 Hz, press press left key). Within a trial stimulus duration was 90 ms, total trial duration was 1300ms. In total 45 trials were presented (22 trials 1000 Hz and 23 trials 1050 Hz). Reaction time and total errors were recorded.

Tone Sequence

The task Tone Sequence addresses auditory processing of tones on a slightly more complex level than in Pitch Differentiation. Within a trial two tones (high tone/1200 Hz/duration 50 ms; low tone/800 Hz/duration 50 ms) were presented successively with a varying interstimulus interval (ISI, 25 ms, 50ms, 100 ms, 150 ms, 200 ms, 300 ms, 600 ms, 1200 ms, 2400 ms) resulting in a tone-sequence. A total of 108 trials was presented in a randomized order. Tone-sequences were either "low-high" (54 trials) or "high-low" (54 trials). Each type of ISI was presented 6 times for low-high and 6 times for high-low. Children had to indicate the kind of sequence via button press: left button press for "low-high" and right button press for high-low. Reaction time and total errors were recorded. The Paradigm followed the paradigm used by Ahissar and colleagues [64]

Syllables

The task *Syllables* was used to investigate auditory performance with linguistic content. Children had to differentiate two acoustically presented syllables *da* and *ga*. Syllable material was kindly provided by Schulte-Körne and colleagues. Synthesized speech stimuli were created by a Klatt synthesiser Klatt, 1980 , the standard stimulus was da and the deviant was ga. Both stimuli had a voice onset time of 31 ms [65]. Subjects had to indicate, which syllable they heard via button press (*da*/left button and *ga*/right button). Within a trial stimulus duration was 220 ms, with an intertrialinterval of 1080ms. In total, 45 trials were presented, 23 *ga* and 22 *da*. Reaction time and total errors were recorded.

S2.3 Visual Performance

We used several tasks with different visual stimulus material to address the magnocellular and parvocellular processing pathways of the visual system.

Parvocellular Visual Processing: Pattern Sequence

Pattern Sequence addresses the parvocellular cell system or visual *What*-system. The parvocellular cell system is responsible for the detection of contrast [66]. In *Pattern Sequence*, the children saw two visual stimuli, which were successively presented in the middle of the computer screen (a circle with a pattern of vertical gratings and a white circle). The children indicated the kind of sequence by pressing a key (sequence empty circle – grating pattern / right button; sequence grating pattern – white circle / left button). Each sequence was presented 25 times, resulting in a total trial number of 50, the presentation of the respective sequence type was randomized. Within a trial each stimulus was presented for 300 ms with an ISI of 500 ms between the stimuli and an intertrialinterval of 750 ms. The reaction time and the absolute number of errors were recorded.

Coherent Motion

Coherent Motion was a task to address the magnocellular cell system visual *Where*-system. Subjects viewed a cloud of randomly moving dots. After 1000 ms either 20, 40 or 60 percent of the dots started to move for 420 ms coherently in the same direction. The subject had to indicate the onset and the direction of the coherent motion detection via button press (left button/left movement, right button/right movement). The program was kindly provided by Schulte-Körne and colleagues (compare [67]. 150 trials were presented with 35 trials for each condition (20% left or right, 40% left or right, 60 percent left or right). The direction of motion and the level of coherent motion were presented randomly. The reaction time and the absolute number of errors were recorded.

Coherent Color

Coherent Color was set up quiet similar to *Coherent Motion.* Yet *Coherent Color* is supposed to address the visual *What*-system acting as a control task by activating the parvocellular cell system. Subjects viewed a cloud of randomly moving dots. After 1000 ms either 20, 40 or 60 percent changed their color from white to yellow (random order of level of coherent color). During the whole time all dots moved around randomly. The children had to indicated the time of color change via button press. The program was kindly made available by Schulte-Körne and colleagues [67]. 75 trials were presented, with 25 trials per condition (20, 40, 60%). Reaction time and total errors were recorded.

Moving and Stationary Gratings

Two experimental conditions (stimuli: stationary/non-moving and moving sine wave vertical and horizontal gratings) were applied, on order to trigger activation of different parts of the visual system. Stationary visual stimuli should evoke activity basically in the parvocellular system, moving visual stimuli should evoke activity in the magnocellular system (see[68] for details regarding stimulus material). Forty non-moving stationary horizontal sine wave grating stimuli were presented for 1100 ms each, followed by an interstimulus interval of 900 ms, during which subjects fixated on a cross appearing on a black computer screen. Subjects were instructed to press a button to indicate their appearance of the stimulus. 120 vertical sine wave grating stimuli were presented for 600 ms. After this time, the sine wave pattern started to move in 6 different conditions (either to the left or to the right, movement to each side in three different velocities (slow / 2 degrees per second, medium / 8 degrees per second, fast/ 16 degress per second) resulting in six conditions. Each condition (e.g. movement to the left in slow speed) was presented twenty times. Duration of movement was 500 ms. After this, the stimulus was cleared from the screen and the subject fixated on a cross appearing on a black computer screen for an

interstimulus interval (ISI, random duration between 900-1000 ms). Reaction time and error rates were recorded.

Mental Rotation of Visual Stimuli (Letters and Objects)

An excellent opportunity to test the functional coordination view is offered by mental-rotation tasks [69]. Mental-rotation tasks have components of both spatial processing and of matching transformed images. In the mental-rotation task two items are presented in succession. This task was developed following[69]. Participants are asked to decide whether these items are the same or different. The stimuli consisted of two letters (k and F) and two graphical forms and their respective mirror images of the letters and forms. They were presented in five different orientations. Each stimulus appeared individually at the centre of a screen against a white background. Colour of the stimuli was black. Trials were presented in random order kept constant across subjects. On each trial, a central fixation point (500 ms) preceded target presentation. The first target was presented for 1500 ms, then the computer screen was cleared and the second stimulus appeared and was presented for 5000 ms. After this time the computer screen was cleared. Participants responded by pressing the left button of a computer mouse in case the letter was the same and the right mouse button, when the stimuli were mirrored images. The inter-trial interval was fixed at 1200 ms. Twenty pairs of letters (10 standard orientation, 10 mirrored) and twenty pairs of shapes (10 standard orientation, 10 mirrored) where presented. The first target always was in standard orientation. The second target was either a rotated or a mirrored version. Subjects had to indicate via button press, whether the second target was a rotated but not mirrored (left button) or rotated and also mirrored (right button). Reaction time and total errors were recorded.

S2.4 Cerebellar Performance

Tasks to operationalize cerebellar performance in the form of sensorimotor processing were taken from the NEPSY [70]. The subtest *Fingertip* is a timed subtest with two parts. The first part is designed to assess the child's finger dexterity and motor speed. The second part is used to assess rapid motor programming. The child copies a series of finger motions demonstrated by the examiner as quickly as possible. The total time for performing all required movements in all parts is recorded.

In the *Statue* test, the child is required to stand still in a position as a "statue" holding a flag over a 75-sec interval. At pre-set intervals distractors are introduced (e.g. the examiner dropping a pen). For each 5-sec interval, the child is awarded 2 points for lack of inappropriate responses, and 1 point for one inappropriate response. The maximum score is 30. This subtest is designed to assess motor persistence and inhibition. The child is asked to maintain a body position with eyes closed during a 75-second period and to inhibit the impulse to respond to sound distracters.

S3. *Results*

S3.1 Auditory Performance

Table S2. auditory performance regarding different ISIs

	NT mean (*SD*)	DYSL mean (*SD*)	statistics	*p*-value
n	21	20		
TS_rct_25ms	1165.08 (454.08)	1052.38 (347.00)	t(37.27)=0.89	$p = 1.000$
TS_rct_50ms	1128.39 (372.51)	1168.67 (507.41)	t(34.80)=–0.29	$p = 1.000$
TS_rct_100ms	1088.10 (507.10)	1075.65 (429.04)	t(38.48)=0.08	$p = 1.000$
TS_rct_150ms	1004.20 (479.20)	1002.16 (434.72)	t(38.91)=0.01	$p = 1.000$
TS_rct_200ms	1107.34 (515.34)	1015.95 (461.58)	t(38.86)=0.60	$p = 1.000$
TS_rct_300ms	1087.71 (393.57)	1091.22 (522.97)	t(35.28)=–0.02	$p = 1.000$
TS_rct_600ms	1008.29 (365.29)	1076.14 (445.40)	t(36.80)=–0.53	$p = 1.000$
TS_rct_1200ms	891.07 (343.63)	893.16 (415.86)	t(36.92)=–0.02	$p = 1.000$
TS_rct_2400ms	1088.10 (507.10)	1075.65 (429.04)	t(38.48)=0.08	$p = 1.000$
TS_err_25ms	0.95 (1.20)	2.80 (1.33)	t(38.13)=–4.65	$p < 0.001$
TS_err_50ms	0.79 (0.94)	2.43 (1.40)	t(33.12)=–4.38	$p < 0.001$
TS_err_100ms	0.62 (0.99)	2.43 (1.28)	t(35.71)=–5.04	$p < 0.001$
TS_err_150ms	0.55 (0.63)	2.88 (1.60)	t(24.55)=–6.08	$p < 0.001$
TS_err_200ms	0.76 (1.15)	2.33 (1.66)	t(33.65)=–3.50	$p < 0.001$
TS_err_300ms	0.71 (0.89)	2.60 (1.60)	t(29.36)=–4.63	$p < 0.001$
TS_err_600ms	0.57 (0.88)	2.43 (1.68)	t(28.47)=–4.39	$p < 0.001$
TS_err_1200ms	0.48 (1.11)	2.75 (1.83)	t(31.05)=–4.78	$p < 0.001$
TS_err_2400ms	0.62 (0.99)	2.43 (1.28)	t(35.71)=–5.04	$p < 0.001$

NT: neurotypical normal reading children, DYSL: children with dyslexia, TS: tone sequence, rct: reaction time, err: errors

S3.2 Fisher's exact test

Table S3: Results of two-sided Fisher's exact test, comparison whether the percentage of individuals with deviations differs statistically between the groups

Index or subtest	1 *SD*	1.65 *SD*
Indices		
index phon	$p < 0.001$	p <0 .001
index RAN	$p = 0.001$	$p = 0.021$
index PWM	$p = 0.054$	$p = 0.232$
index auditory total	$p = 0.480$	$p = 0.232$
index auditory error	$p < 0.001$	$p = 0.005$
index auditory rct	$p = 1.000$	$p = 0.488$
index vis magno total	n.n.	n.n.
index vis magno rct	n.n.	n.n.
index vis magno error	n.n.	n.n.
index vis parvo total	n.n.	n.n.
index vis parvo rct	n.n.	n.n.
index vis parvo error	n.n.	n.n.
index cerebellar	$p = 0.130$	$p = 0.343$
Phonological Awareness		
Pseudo Segm	$p = 0.159$	$p = 0.184$
Vow Subst	$p = 1.000$	$p = 0.454$
Phon Swap	$p = 0.697$	$p = 0.663$

Sound Cat	$p = 0.005$	$p < 0.001$
Word Revers	$p = 0.025$	$p = 0.009$
1 syl PW rct	$p < 0.001$	$p < 0.001$
1 syl PW error	$p = 0.025$	$p = 0.025$
3 syl PW rct	$p < 0.001$	$p < 0.001$
3 syl PW error	$p = 0.011$	$p = 0.009$
Phonological recoding in working memory		
digit repetition	$p = 0.067$	$p = 0.232$
Phonological recoding with access to the semantic lexicon		
RAN obj b/w rct	$p = 0.003$	$p = 0.006$
RAN b/w obj error	$p = 0.184$	$p = 0.184$
RAN colour obj rct	$p = 0.043$	$p = 0.020$
RAN colour obj error	$p = 0.343$	$p = 0.343$
RAN letter rct	$p < 0.001$	$p = 0.009$
RAN letter error	$p = 0.505$	$p = 0.606$
Auditory Processing		
single tone rct	$p = 0.343$	$p = 0.488$
tonesequence rct	$p = 1.000$	$p = 1.000$
syllables rct	$p = 0.663$	n.n.
single tone error	$p < 0.001$	$p = 0.003$
tonesequence error	$p < 0.001$	$p < 0.001$
syllables errors	$p = 0.277$	$p = 0.045$
TS_rct_25ms	$p = 0.606$	$p = 0.488$
TS_rct_50ms	$p = 1.000$	$p > 1.000$
TS_rct_100ms	$p = 1.000$	$p > 1.000$
TS_rct_150ms	$p = 0.663$	$p = 1.000$
TS_rct_200ms	$p = 0.663$	$p = 0.606$
TS_rct_300ms	$p = 1.000$	$p = 0.606$
TS_rct_600ms	$p = 0.697$	$p = 0.343$
TS_rct_1200ms	$p = 1.000$	$p = 0.606$
TS_rct_2400ms	$p = 1.000$	$p = 1.000$
TS_err_25ms	$p = 0.004$	$p = 0.004$
TS_err_50ms	$p < 0.001$	$p = 0.004$
TS_err_100ms	$p < 0.001$	$p < 0.001$
TS_err_150ms	$p < 0.001$	$p < 0.001$
TS_err_200ms	$p < 0.001$	$p = 0.001$
TS_err_300ms	$p < 0.001$	$p < 0.001$
TS_err_600ms	$p < 0.001$	$p < 0.001$
TS_err_1200ms	$p < 0.001$	$p < 0.001$
TS_err_2400ms	$p < 0.001$	$p < 0.001$
Visual Processing		
Magnocellular System		
CM rct	$p = 0.048$	$p = 0.488$
CM error	$p = 0.606$	$p = 1.000$
MG rct	$p = 0.343$	$p = 1.000$
MG error	$p = 0.719$	$p = 0.663$
Parvocellular System		
CC rct	$p = 0.184$	$p = 1.000$

CC error	n.n.	n.n.
PS rct	*p* = 1.000	*p* = 1.000
PS error	*p* = 0.003	*p* = 0.032
StG rct	*p* = 1.000	*p* = 1.000
StG error	*p* = 1.000	*p* = 1.000
Other visual Control Tasks		
Ro rct	*p* = 1.000	*p* = 1.000
Ro error	*p* = 0.719	*p* = 1.000
CM rct 1	*p* = 0,343	*p* = 1.000
CM rct 2	*p* = 0,232	*p* = 0.488
CM rct 3	*p* = 0,184	*p* = 1.000
CM rct 4	*p* = 0.343	*p* = 1.000
CM rct 5	*p* = 0.107	*p* = 1.000
CM rct 6	*p* = 0.184	*p* = 0.488
CM error 1	*p* = 0.663	*p* = 1.000
CM error 2	*p* = 1.000	*p* = 1.000
CM error 3	*p* = 0.606	*p* = 1.000
CM error 4	*p* = 1.000	*p* = 1.000
CM error 5	*p* = 1.000	*p* = 1.000
CM error 6	*p* = 0.606	*p* = 1.000
MG rct 1	*p* = 0.663	*p* = 0.488
MG rct 2	*p* = 1.000	*p* = 0.488
MG rct 3	*p* = 0.606	*p* = 1.000
MG error 1	*p* = 0.208	*p* = 0.343
MG error 2	*p* = 0.697	*p* = 1.000
MG error 3	*p* = 0.454	*p* = 0.343
Cerebellar Processing		
tapping	*p* = 0.015	*p* = 0.410
statue	*p* = 0.484	*p* = 0.481

S3.3 Z-Score Performance in Indices

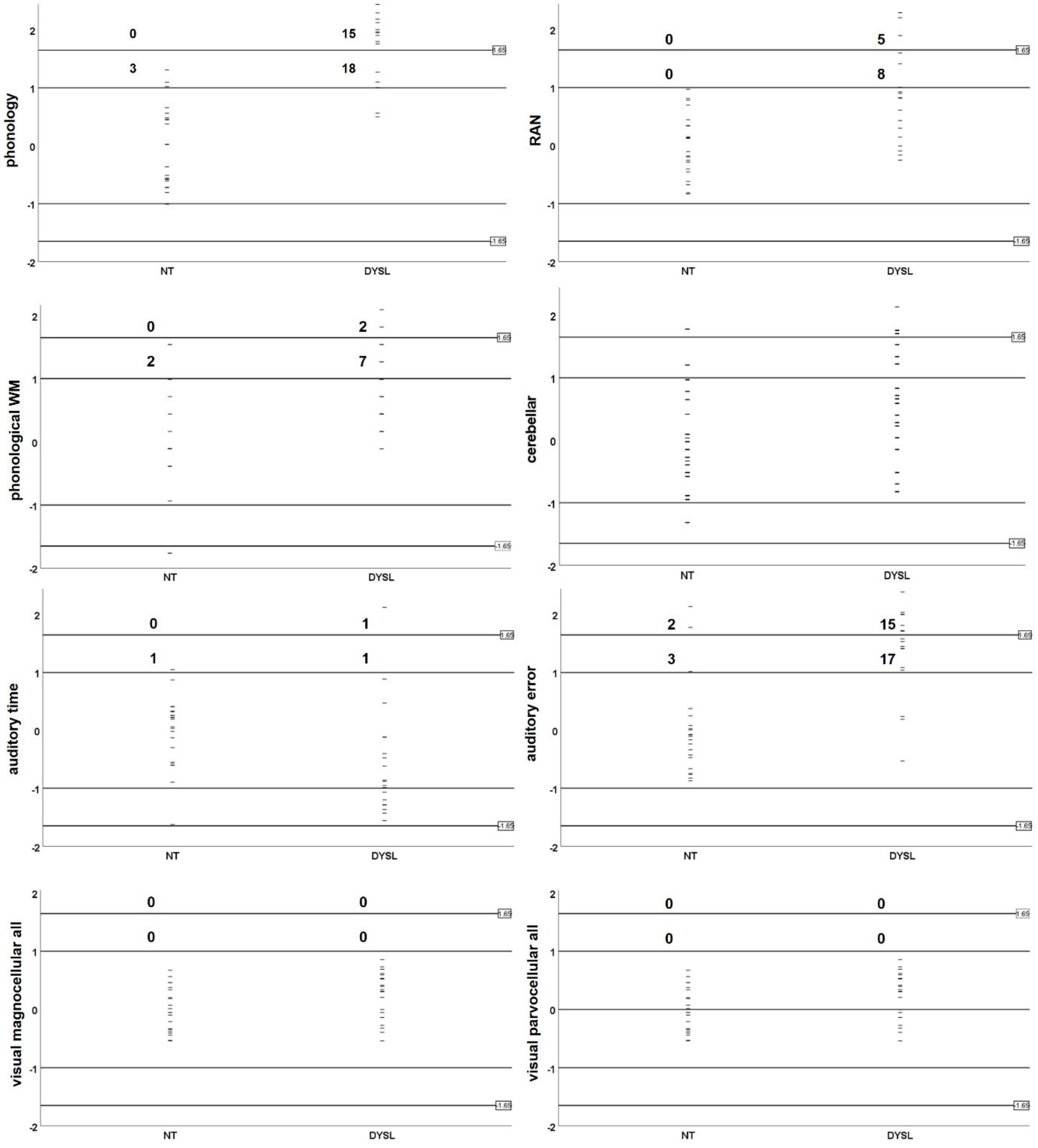

Figure S1: *z*-sores of performance in the different indices, NT: neurotypical readers, Dysl: dyslexic readers, number above the *SD*-thresholds: number of individuals with deviating performance in the respective index, y-axis: unit in *z*-scores (mean: 0, *SD*: 1), lines as definitions for outliers are at 1 and 1.65 *SD*, RAN: rapid automatized naming, WM: working memory

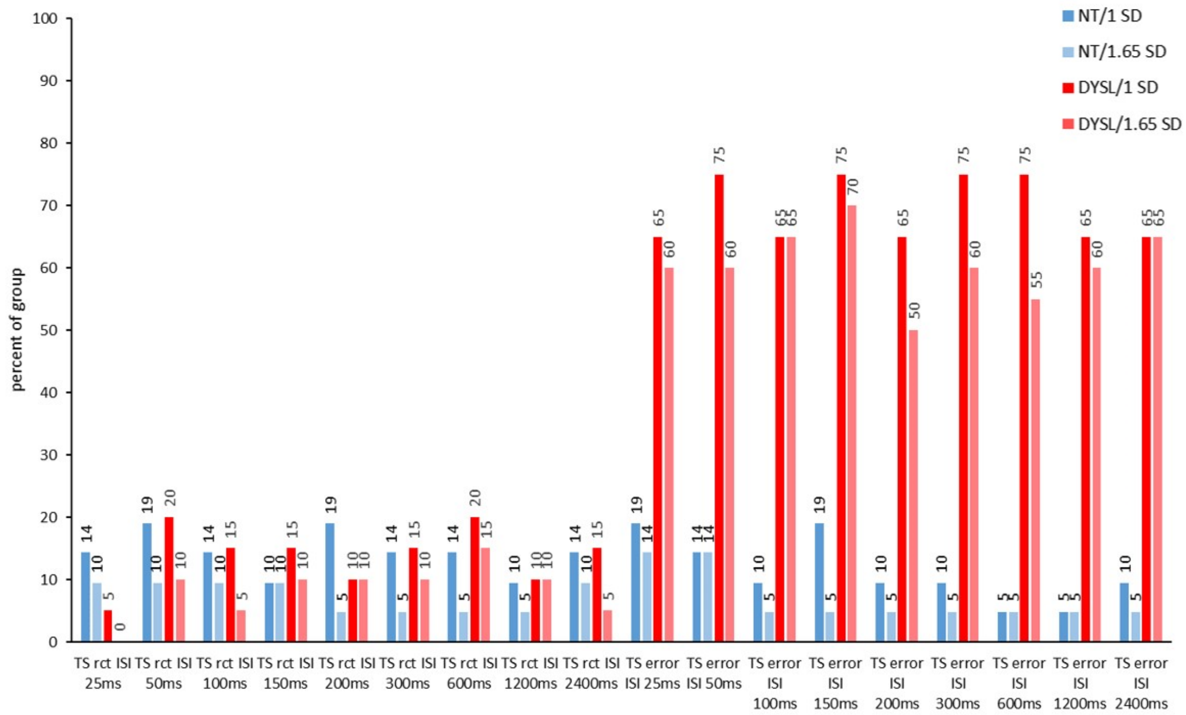

S3.4. Additional figures on subtestlevel

Figure S2: deviating performance / auditory subtests; performance within tone sequences for different ISIs; NT: neurotypical normal reading children, DYSL: children with dyslexia, rct: reaction time, TS: tone sequence, ISI: interstimulus interval, ms: milliseconds;

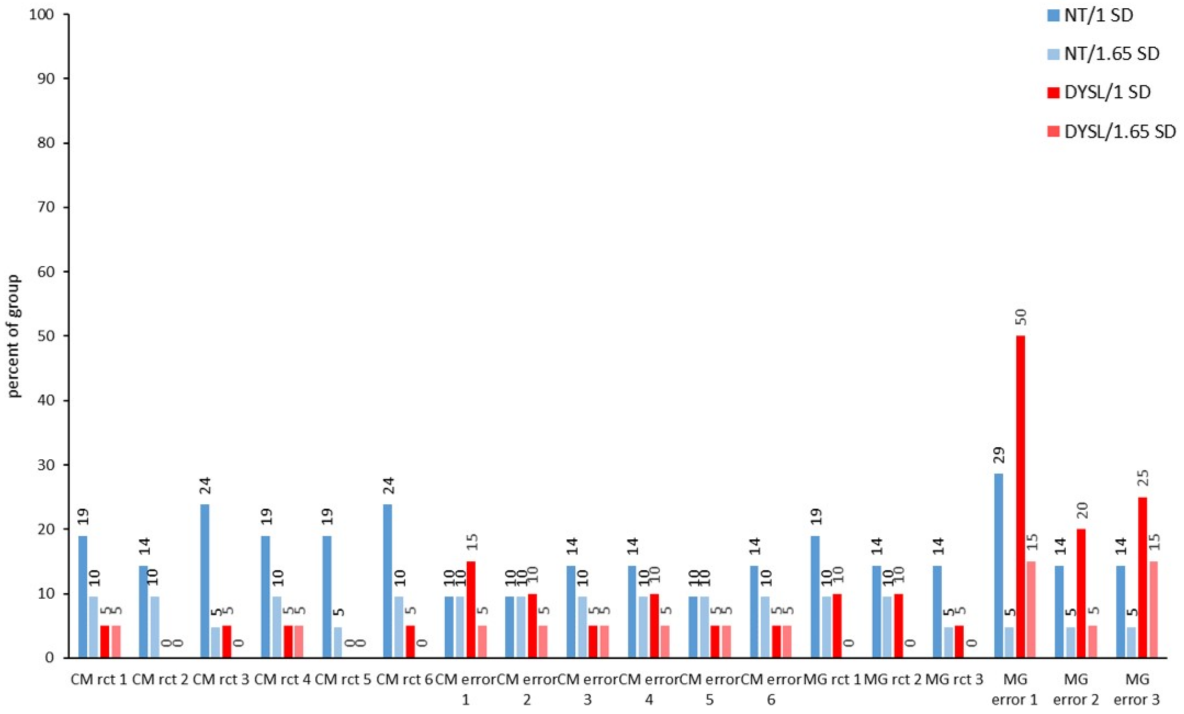

Figure S3: deviating performance / visual subtests at different speeds; NT: neurotypical normal reading children, DYSL: children with dyslexia, rct: reaction

time, err: errors, MG: Moving Gratings, CM: Coherent Motion, CC: Coherent Color, PS: Pattern Sequence, StG: Stationary Gratings, Ro: Mental Rotation

S3.5 Additional figures on the individual level presented in heatmaps for 1.65 SD

NT/1.65 SD	40	42	44	47	49	51	55	57	59	62	66	67	70	74	75	78	79	80	81	85	95
index phon	0	0	0	0	0	0	0	0	0	0	0	0	0	0	0	0	0	0	0	0	0
index RAN	0	0	0	0	0	0	0	0	0	0	0	0	0	0	0	0	0	0	0	0	0
Index PWM	0	0	0	0	0	0	0	0	0	0	0	0	0	0	0	0	0	0	0	0	0
index auditory total	0	0	0	0	0	0	0	0	0	0	0	0	0	0	0	0	0	0	0	0	0
index auditory rct	0	0	0	0	0	0	0	0	0	0	0	0	0	0	0	0	0	0	0	0	0
index auditory error	1	0	0	0	0	0	0	0	0	0	0	0	1	0	0	0	0	0	0	0	0
index vis magno total	0	0	0	0	0	0	0	0	0	0	0	0	0	0	0	0	0	0	0	0	0
index vis magno rct	0	0	0	0	0	0	0	0	0	0	0	0	0	0	0	0	0	0	0	0	0
index vis magno error	0	0	0	0	0	0	0	0	0	0	0	0	0	0	0	0	0	0	0	0	0
index vis parvo total	0	0	0	0	0	0	0	0	0	0	0	0	0	0	0	0	0	0	0	0	0
index vis parvo rct	0	0	0	0	0	0	0	0	0	0	0	0	0	0	0	0	0	0	0	0	0
index vis parvo error	0	0	0	0	0	0	0	0	0	0	0	0	0	0	0	0	0	0	0	0	0
index cerebellar	0	0	0	0	0	1	0	0	0	0	0	0	0	0	0	0	0	0	0	0	0

DYSL/1.65 SD	37	38	39	43	46	52	58	63	64	65	71	76	86	98	100	101	114	117	118	119
index phon	0	0	1	1	1	0	0	1	1	1	1	1	1	1	1	1	1	0	1	1
index RAN	0	0	1	1	0	0	0	0	0	1	0	0	0	0	1	0	1	0	0	0
Index PWM	0	0	0	0	0	0	0	0	0	0	0	0	0	0	0	0	1	1	0	0
index auditory total	0	0	0	0	0	0	1	0	0	0	0	0	0	0	0	0	0	1	0	0
index auditory rct	0	0	0	0	0	0	0	0	0	0	0	0	0	0	0	0	0	1	0	0
index auditory error	0	1	1	0	0	0	1	1	1	0	0	0	1	0	1	1	0	0	1	1
index vis magno total	0	0	0	0	0	0	0	0	0	0	0	0	0	0	0	0	0	0	0	0
index vis magno rct	0	0	0	0	0	0	0	0	0	0	0	0	0	0	0	0	0	0	0	0
index vis magno error	0	0	0	0	0	0	0	0	0	0	0	0	0	0	0	0	0	0	0	0
index vis parvo total	0	0	0	0	0	0	0	0	0	0	0	0	0	0	0	0	0	0	0	0
index vis parvo rct	0	0	0	0	0	0	0	0	0	0	0	0	0	0	0	0	0	0	0	0
index vis parvo error	0	0	0	0	0	0	0	0	0	0	0	0	0	0	0	0	0	0	0	0
index cerebellar	1	1	0	0	1	0	0	0	0	0	0	0	0	0	0	0	0	0	0	0

Figure S4: deviating individual performance on the indexlevel (1.65 SD), NT: neurotypical normal reading children, DYSL: children with dyslexia, numbers in the head row depict the individual subject, phon: phonological awareness, RAN: rapid automatized naming, PWM: phonological working memory, rct: reaction time, magno: magnocellular, parvo: parvocellular

NT/1.65 SD	40	42	44	47	49	51	55	57	59	62	66	67	70	74	75	78	79	80	81	85	95
pseudo segm	0	0	0	0	0	0	0	0	0	0	0	0	0	0	0	0	1	0	0	0	0
vow subst	0	0	0	0	0	0	0	1	0	0	0	0	1	0	0	0	0	1	0	0	0
phon swap	0	0	0	0	0	0	0	0	0	0	0	0	0	0	0	0	1	1	0	0	0
sound cat	0	0	0	0	0	0	0	0	0	0	0	0	1	0	0	0	0	1	0	0	0
word revers	0	0	0	0	0	0	0	0	0	0	0	0	1	0	0	0	0	0	0	0	0
1 syl PW rct	0	0	1	0	1	0	0	0	0	0	0	0	0	0	0	0	0	0	0	0	0
1 syl PW error	0	0	1	0	0	0	0	0	0	0	1	0	0	0	1	0	0	0	1	0	0
3 syl PW rct	0	0	1	0	0	0	0	0	0	0	0	0	0	0	0	0	0	0	0	0	0
3 syl PW error	0	0	0	0	0	0	0	0	0	0	1	0	0	0	0	0	0	0	0	0	0
digit repetition	0	0	0	0	0	0	0	0	0	0	0	0	0	0	0	0	0	0	0	0	0
RAN obj b/w rct	0	0	1	0	1	0	0	0	0	0	0	0	0	0	0	0	0	0	0	0	0
RAN b/w obj error	0	0	0	0	0	1	0	0	0	0	0	0	0	0	0	0	0	0	0	0	0
RAN colour obj rct	0	0	0	0	1	0	0	0	0	0	0	0	0	0	0	0	0	0	0	0	0
RAN coulor obj error	0	0	0	0	0	0	0	0	0	0	0	0	0	0	1	0	0	0	0	0	0
RAN letter rct	0	0	1	0	0	0	0	0	0	0	0	0	0	0	0	0	0	0	0	0	0
RAN letter error	0	0	0	0	0	1	0	0	0	0	0	0	0	0	0	0	0	0	0	0	0

DYSL/1.65 SD	37	38	39	43	46	52	58	63	64	65	71	76	86	98	100	101	114	117	118	119
pseudo segm	0	0	0	0	0	0	0	0	1	0	0	0	1	0	1	0	0	0	1	0
vow subst	1	1	1	0	0	0	0	0	1	0	0	0	1	0	0	0	0	0	0	0
phon swap	0	0	0	0	0	0	0	0	1	0	0	0	0	0	1	0	0	0	1	0
sound cat	0	1	1	1	0	0	1	0	1	1	0	1	1	0	1	0	1	0	1	1
word revers	0	0	1	0	1	0	0	0	1	1	1	0	0	0	1	0	0	0	1	1
1 syl PW rct	0	1	1	0	1	1	0	1	1	1	1	1	1	1	1	1	1	1	1	0
1 syl PW error	0	0	0	1	1	1	0	1	1	0	1	0	1	0	1	1	0	0	1	1
3 syl PW rct	0	0	1	0	1	0	0	1	0	1	1	1	1	1	1	1	1	1	1	0
3 syl PW error	0	0	0	1	0	0	0	1	1	1	0	0	0	0	1	1	0	0	1	1
digit repetition	0	0	0	0	0	0	0	0	0	0	0	0	0	0	0	0	1	1	0	0
RAN obj b/w rct	1	0	1	0	0	0	0	0	0	1	1	1	1	1	1	1	1	0	0	0
RAN b/w obj error	0	0	0	1	1	0	0	0	0	0	0	0	0	0	0	0	1	0	0	1
RAN colour obj rct	0	0	1	0	0	0	0	0	0	1	0	1	0	0	1	1	1	1	0	0
RAN coulor obj error	0	0	0	1	0	0	0	0	0	0	0	0	0	1	1	0	0	0	0	0
RAN letter rct	0	0	1	1	1	0	0	0	0	1	1	0	0	1	1	0	1	0	0	0
RAN letter error	0	0	0	0	0	0	1	0	0	0	0	0	0	0	0	0	1	0	0	0

Figure S5: deviating individual performance / phonological subtests (1.65 SD); NT: neurotypical normal reading children, DYSL: children with dyslexia, numbers in the head row depict the individual subject, rct: reaction time, err: errors, Phon Mean: mean of the phonological subtests for phonological processing in an inner sense, syl: syllables, PW: pseudowords, RAN: rapid naming, b/w: black and white, obj: object

NT/1.65 SD	40	42	44	47	49	51	55	57	59	62	66	67	70	74	75	78	79	80	81	85	95
single tone rct	0	0	0	0	0	0	0	0	0	0	0	0	0	0	0	0	0	0	0	0	0
tonesequence rct	0	1	0	0	0	0	0	0	0	1	0	0	0	0	0	0	0	0	0	0	0
syllables rct	0	0	0	0	0	0	0	0	0	0	0	0	0	0	0	0	0	0	0	0	0
single tone error	1	1	0	0	0	0	0	0	0	0	0	0	0	0	0	0	0	0	0	0	0
tonesequece error	0	0	0	0	0	0	0	0	0	0	0	0	1	0	0	0	0	0	0	0	0
syllables errors	1	0	0	0	0	0	0	0	0	0	0	0	0	0	0	0	0	0	0	0	0

DYSL/1.65 SD	37	38	39	43	46	52	58	63	64	65	71	76	86	98	100	101	114	117	118	119
single tone rct	0	0	0	0	0	0	0	0	0	0	0	0	0	0	0	0	0	1	0	0
tonesequence rct	0	0	0	0	0	0	0	0	0	0	0	0	0	1	0	0	0	1	0	0
syllables rct	0	0	0	0	0	0	0	0	0	0	0	0	0	0	0	0	0	0	0	0
single tone error	0	1	1	0	0	1	1	0	0	1	0	0	1	1	1	1	0	1	1	0
tonesequece error	0	1	1	0	1	1	1	1	1	0	1	0	1	1	1	0	0	1	1	1
syllables errors	1	1	0	0	0	0	1	1	1	0	0	0	0	0	0	0	0	0	0	1

Figure S6: deviating individual performance /auditory subtests (1.65 SD); NT: neurotypical normal reading children, DYSL: children with dyslexia, numbers in the head row depict the individual subject, rct: reaction time, err: errors, TS: tone sequence ISI: interstimulus interval

NT/1 SD	40	42	44	47	49	51	55	57	59	62	66	67	70	74	75	78	79	80	81	85	95
TS rct ISI 25ms	0	1	0	0	0	0	0	0	0	1	0	0	0	0	1	0	0	0	0	0	0
TS rct ISI 50ms	0	1	0	0	0	0	0	0	1	1	0	0	0	0	0	0	0	0	1	0	0
TS rct ISI 100ms	0	1	0	0	0	0	0	1	0	1	0	0	0	0	0	0	0	0	0	0	0
TS rct ISI 150ms	0	1	0	0	0	0	0	0	0	1	0	0	0	0	0	0	0	0	0	0	0
TS rct ISI 200ms	0	1	0	0	0	0	0	0	0	1	0	0	0	0	0	0	0	0	1	0	1
TS rct ISI 300ms	0	0	0	0	1	0	0	0	0	1	0	0	1	0	0	0	0	0	0	0	0
TS rct ISI 600ms	0	0	0	0	1	0	0	0	0	1	0	0	0	0	0	0	0	0	1	0	0
TS rct ISI 1200ms	1	0	0	0	0	0	0	0	0	0	0	0	0	0	0	0	0	0	1	0	0
TS rct ISI 2400ms	0	1	0	0	0	0	0	1	0	1	0	0	0	0	0	0	0	0	0	0	0
TS error ISI 25ms	1	0	0	0	0	0	0	0	0	1	0	0	1	0	1	0	0	0	0	0	0
TS error ISI 50ms	0	0	0	0	0	0	0	0	0	1	0	0	1	0	0	0	0	0	0	1	0
TS error ISI 100ms	0	0	0	0	0	0	0	0	0	1	0	0	1	0	0	0	0	0	0	0	0
TS error ISI 150ms	1	0	0	0	0	0	0	0	0	0	0	0	1	0	1	0	0	1	0	0	0
TS error ISI 200ms	0	0	0	0	0	0	0	0	0	1	0	0	1	0	0	0	0	0	0	0	0
TS error ISI 300ms	0	0	0	0	0	0	0	0	0	1	0	0	1	0	0	0	0	0	0	0	0
TS error ISI 600ms	0	0	0	0	0	0	0	0	0	0	0	0	1	0	0	0	0	0	0	0	0
TS error ISI 1200ms	0	0	0	0	0	0	0	0	0	0	0	0	1	0	0	0	0	0	0	0	0
TS error ISI 2400ms	0	0	0	0	0	0	0	0	0	1	0	0	1	0	0	0	0	0	0	0	0

DYSL/1 SD	37	38	39	43	46	52	58	63	64	65	71	76	86	98	100	101	114	117	118	119
TS rct ISI 25ms	0	0	0	0	0	0	0	0	0	0	0	0	0	1	0	0	0	0	0	0
TS rct ISI 50ms	1	1	0	0	0	0	0	0	0	0	0	0	0	1	0	0	0	1	0	0
TS rct ISI 100ms	0	0	0	0	0	0	0	0	0	0	0	0	1	1	0	0	0	1	0	0
TS rct ISI 150ms	0	1	0	0	0	0	0	0	0	0	0	0	0	1	0	0	0	1	0	0
TS rct ISI 200ms	0	0	0	0	0	0	0	0	0	0	0	0	0	1	0	0	0	1	0	0
TS rct ISI 300ms	0	0	0	0	0	0	0	0	0	0	0	0	0	1	0	0	0	1	1	0
TS rct ISI 600ms	0	0	0	0	0	0	0	0	0	0	0	0	0	1	0	0	1	1	0	1
TS rct ISI 1200ms	0	0	0	0	0	0	0	0	0	0	0	0	0	1	0	0	0	1	0	0
TS rct ISI 2400ms	0	0	0	0	0	0	0	0	0	0	0	0	1	1	0	0	0	1	0	0
TS error ISI 25ms	0	1	1	0	0	0	1	1	1	1	1	0	1	1	1	0	0	1	1	1
TS error ISI 50ms	0	1	1	0	1	0	1	1	1	1	1	1	1	1	1	0	0	1	1	1
TS error ISI 100ms	0	1	1	0	1	1	1	1	1	0	1	0	1	1	1	0	0	1	0	1
TS error ISI 150ms	0	1	1	0	1	1	1	1	1	0	1	1	1	1	1	0	0	1	1	1
TS error ISI 200ms	0	1	1	0	1	1	1	1	1	1	1	0	1	0	1	0	0	1	1	0
TS error ISI 300ms	0	1	1	0	1	1	1	1	1	1	1	0	1	1	1	0	0	1	1	1
TS error ISI 600ms	0	1	1	0	1	1	1	1	1	1	1	0	1	1	1	0	0	1	1	1
TS error ISI 1200ms	0	1	1	0	1	1	1	1	1	0	1	0	1	1	1	0	0	1	0	1
TS error ISI 2400ms	0	1	1	0	1	1	1	1	1	0	1	0	1	1	1	0	0	1	0	1

NT/1.65 SD	40	42	44	47	49	51	55	57	59	62	66	67	70	74	75	78	79	80	81	85	95
TS rct ISI 25ms	0	0	0	0	0	0	0	0	0	1	0	0	0	0	1	0	0	0	0	0	0
TS rct ISI 50ms	0	1	0	0	0	0	0	0	0	1	0	0	0	0	0	0	0	0	0	0	0
TS rct ISI 100ms	0	1	0	0	0	0	0	0	0	1	0	0	0	0	0	0	0	0	0	0	0
TS rct ISI 150ms	0	1	0	0	0	0	0	0	0	1	0	0	0	0	0	0	0	0	0	0	0
TS rct ISI 200ms	0	0	0	0	0	0	0	0	0	1	0	0	0	0	0	0	0	0	0	0	0
TS rct ISI 300ms	0	0	0	0	0	0	0	0	0	1	0	0	0	0	0	0	0	0	0	0	0
TS rct ISI 600ms	0	0	0	0	0	0	0	0	0	1	0	0	0	0	0	0	0	0	0	0	0
TS rct ISI 1200ms	1	0	0	0	0	0	0	0	0	0	0	0	0	0	0	0	0	0	0	0	0
TS rct ISI 2400ms	0	1	0	0	0	0	0	0	0	1	0	0	0	0	0	0	0	0	0	0	0
TS error ISI 25ms	0	0	0	0	0	0	0	0	0	1	0	0	1	0	1	0	0	0	0	0	0
TS error ISI 50ms	0	0	0	0	0	0	0	0	0	1	0	0	1	0	0	0	0	0	0	1	0
TS error ISI 100ms	0	0	0	0	0	0	0	0	0	0	0	0	1	0	0	0	0	0	0	0	0
TS error ISI 150ms	0	0	0	0	0	0	0	0	0	0	0	0	0	0	1	0	0	0	0	0	0
TS error ISI 200ms	0	0	0	0	0	0	0	0	0	0	0	0	1	0	0	0	0	0	0	0	0
TS error ISI 300ms	0	0	0	0	0	0	0	0	0	0	0	0	1	0	0	0	0	0	0	0	0
TS error ISI 600ms	0	0	0	0	0	0	0	0	0	0	0	0	1	0	0	0	0	0	0	0	0
TS error ISI 1200ms	0	0	0	0	0	0	0	0	0	0	0	0	1	0	0	0	0	0	0	0	0
TS error ISI 2400ms	0	0	0	0	0	0	0	0	0	0	0	0	1	0	0	0	0	0	0	0	0

DYSL/1.65 SD	37	38	39	43	46	52	58	63	64	65	71	76	86	98	100	101	114	117	118	119
TS rct ISI 25ms	0	0	0	0	0	0	0	0	0	0	0	0	0	0	0	0	0	0	0	0
TS rct ISI 50ms	0	0	0	0	0	0	0	0	0	0	0	0	0	1	0	0	0	1	0	0
TS rct ISI 100ms	0	0	0	0	0	0	0	0	0	0	0	0	0	1	0	0	0	0	0	0
TS rct ISI 150ms	0	0	0	0	0	0	0	0	0	0	0	0	0	1	0	0	0	1	0	0
TS rct ISI 200ms	0	0	0	0	0	0	0	0	0	0	0	0	0	1	0	0	0	1	0	0
TS rct ISI 300ms	0	0	0	0	0	0	0	0	0	0	0	0	0	1	0	0	0	1	0	0
TS rct ISI 600ms	0	0	0	0	0	0	0	0	0	0	0	0	0	1	0	0	1	0	0	1
TS rct ISI 1200ms	0	0	0	0	0	0	0	0	0	0	0	0	0	1	0	0	0	1	0	0
TS rct ISI 2400ms	0	0	0	0	0	0	0	0	0	0	0	0	0	1	0	0	0	0	0	0
TS error ISI 25ms	0	1	1	0	0	0	1	0	1	1	1	0	1	1	1	0	0	1	1	1
TS error ISI 50ms	0	1	1	0	1	0	1	1	1	0	0	0	1	1	1	0	0	1	1	1
TS error ISI 100ms	0	1	1	0	1	1	1	1	1	0	1	0	1	1	1	0	0	1	0	1
TS error ISI 150ms	0	1	1	0	1	1	1	1	1	0	1	0	1	1	1	0	0	1	1	1
TS error ISI 200ms	0	1	1	0	1	1	1	1	1	0	0	0	1	0	1	0	0	0	1	0
TS error ISI 300ms	0	1	1	0	0	1	1	1	1	0	1	0	1	0	1	0	0	1	1	1
TS error ISI 600ms	0	1	1	0	1	1	1	1	1	0	1	0	0	0	1	0	0	1	1	0
TS error ISI 1200ms	0	1	1	0	1	1	1	1	1	0	1	0	1	0	1	0	0	1	0	1
TS error ISI 2400ms	0	1	1	0	1	1	1	1	1	0	1	0	1	1	1	0	0	1	0	1

Figure S7: deviating individual performance / auditory (1 SD and 1.65 SD)/ tonesequences / reaction time and errors depicted for different ISIs; NT: neurotypical normal reading children, DYSL: children with dyslexia, numbers in the head row depict the individual subject, rct: reaction time, err: errors, TS: tone sequence ISI: interstimulus interval

NT/1.65 SD	40	42	44	47	49	51	55	57	59	62	66	67	70	74	75	78	79	80	81	85	95
CM rct	0	0	0	0	0	0	1	0	0	0	1	0	0	0	0	0	0	0	0	0	0
CM error	0	0	0	0	0	0	0	0	0	0	1	0	0	0	0	0	0	0	0	0	1
MG rct	0	0	0	0	0	0	0	0	0	0	0	0	0	1	0	0	0	0	0	0	0
MG error	0	0	1	0	0	0	0	0	0	0	0	0	0	0	0	1	0	0	0	0	0
CC rct	0	0	0	0	1	0	0	0	0	0	0	0	0	1	0	0	0	0	0	0	0
CC error	0	0	0	0	0	0	0	0	0	0	0	0	0	0	0	0	0	0	0	0	0
PS time	1	0	0	0	0	0	0	0	0	0	0	0	0	0	0	0	0	0	0	0	0
PS errors	0	0	1	0	0	0	0	0	0	0	0	0	0	0	0	0	0	0	0	1	0
SG rct	0	0	0	0	0	0	0	0	0	0	0	0	0	1	0	0	0	0	0	0	0
SG error	0	0	0	0	0	0	0	0	0	0	0	0	0	1	0	0	0	0	0	0	0
RO rct	0	0	0	0	0	0	0	0	0	0	0	0	0	0	0	0	0	1	1	0	0
RO error	0	0	1	0	0	0	0	0	0	0	0	0	0	0	0	0	0	0	0	0	0

DYSL/1.65 SD	37	38	39	43	46	52	58	63	64	65	71	76	86	98	100	101	114	117	118	119
CM rct	0	0	0	0	0	0	0	0	0	0	0	0	0	0	0	0	0	0	0	0
CM error	0	0	0	0	1	0	0	0	0	0	0	0	0	0	0	0	0	0	0	0
MG rct	0	0	0	0	0	0	0	0	0	0	0	0	0	0	0	0	0	0	0	0
MG error	0	0	0	0	0	0	1	0	0	0	0	0	0	0	0	1	1	0	0	0
CC rct	0	0	0	0	0	0	0	0	0	0	0	0	0	0	0	1	0	0	0	0
CC error	0	0	0	0	0	0	0	0	0	0	0	0	0	0	0	0	0	0	0	0
PS time	0	0	0	0	0	0	0	0	0	0	0	0	0	0	0	0	0	0	0	0
PS errors	1	0	1	0	0	1	0	0	1	0	1	0	1	0	0	0	0	0	1	1
SG rct	0	0	0	0	0	0	0	0	0	0	0	0	0	0	0	0	0	0	0	0
SG error	0	0	0	0	0	0	0	0	0	0	1	0	0	0	0	0	0	0	0	0
RO rct	0	0	0	0	0	0	0	0	0	0	0	0	0	0	0	0	0	1	0	0
RO error	0	0	0	0	0	0	1	0	0	0	0	0	0	0	0	0	0	0	0	0

Figure S8: deviating individual performance/ visual / subtests (1.65 SD) NT: neurotypical normal reading children, DYSL: children with dyslexia, numbers in the head row depict the individual subject, rct: reaction time, err: errors, CM: Coherent Motion, MG: Moving Gratings, CC: Coherent Color, PS: Pattern Sequence, StG: Stationary Gratings, Ro: Rotation

NT/1 SD	40	42	44	47	49	51	55	57	59	62	66	67	70	74	75	78	79	80	81	85	95
CM rct 1	0	0	0	0	1	0	0	0	0	0	1	0	0	1	0	0	0	1	0	0	0
CM rct 2	0	0	0	0	0	0	0	0	0	0	1	0	0	0	0	0	0	1	0	0	1
CM rct 3	0	0	0	0	0	0	1	0	0	1	1	0	0	1	0	0	0	1	0	0	0
CM rct 4	0	0	0	0	0	0	1	0	0	0	1	0	0	0	0	0	0	1	0	0	1
CM rct 5	0	0	0	0	1	0	1	0	0	0	1	0	0	0	0	0	0	0	0	0	1
CM rct 6	0	0	0	0	0	0	1	0	0	0	1	0	0	1	0	0	0	1	0	0	1
CM error 1	0	0	0	0	0	0	0	0	0	0	1	0	0	0	0	0	0	0	0	0	1
CM error 2	0	0	0	0	0	0	0	0	0	0	1	0	0	0	0	0	0	0	0	0	1
CM error 3	0	0	0	0	0	0	0	0	0	0	1	0	0	0	0	0	0	0	1	0	1
CM error 4	0	0	0	0	0	0	0	0	0	0	1	0	0	0	0	0	0	0	1	0	1
CM error 5	0	0	0	0	0	0	0	0	0	0	1	0	0	0	0	0	0	0	0	0	1
CM error 6	0	0	0	0	0	0	0	0	0	0	1	0	0	0	0	0	0	0	1	0	1
MG rct 1	0	0	0	0	1	0	0	0	0	0	0	0	0	1	0	0	1	0	0	1	0
MG rct 2	0	0	0	0	0	0	0	0	0	0	0	0	0	1	0	0	1	0	0	1	0
MG rct 3	0	0	0	0	1	0	0	0	0	0	0	0	0	1	0	0	1	0	0	0	0
MG error 1	0	1	0	0	0	1	0	0	0	0	0	1	0	0	1	0	0	0	0	1	1
MG error 2	0	0	1	0	0	0	0	0	0	0	0	0	1	1	0	0	0	0	0	0	0
MG error 3	0	0	1	0	0	1	0	0	0	0	0	0	0	0	0	1	0	0	0	0	0

DYSL/1 SD	37	38	39	43	46	52	58	63	64	65	71	76	86	98	100	101	114	117	118	119
CM rct 1	0	0	1	0	0	0	0	0	0	0	0	0	0	0	0	0	0	0	0	0
CM rct 2	0	0	0	0	0	0	0	0	0	0	0	0	0	0	0	0	0	0	0	0
CM rct 3	0	0	1	0	0	0	0	0	0	0	0	0	0	0	0	0	0	0	0	0
CM rct 4	0	0	0	0	0	0	0	0	0	0	0	0	0	0	0	0	0	1	0	0
CM rct 5	0	0	0	0	0	0	0	0	0	0	0	0	0	0	0	0	0	0	0	0
CM rct 6	0	0	0	0	0	0	0	0	0	0	0	0	0	0	0	0	0	1	0	0
CM error 1	0	1	0	0	1	0	0	0	0	0	0	1	0	0	0	0	0	0	0	0
CM error 2	0	0	0	0	1	0	0	0	1	0	0	0	0	0	0	0	0	0	0	0
CM error 3	0	0	0	0	1	0	0	0	0	0	0	0	0	0	0	0	0	0	0	0
CM error 4	0	0	0	0	1	0	0	0	0	1	0	0	0	0	0	0	0	0	0	0
CM error 5	0	0	0	0	1	0	0	0	0	0	0	0	0	0	0	0	0	0	0	0
CM error 6	0	0	0	0	1	0	0	0	0	0	0	0	0	0	0	0	0	0	0	0
MG rct 1	0	0	0	1	0	0	0	0	0	0	0	0	0	0	0	0	0	0	1	0
MG rct 2	0	0	0	1	0	0	0	0	0	0	1	0	0	0	0	0	0	0	0	0
MG rct 3	0	0	0	1	0	0	0	0	0	0	0	0	0	0	0	0	0	0	0	0
MG error 1	1	1	0	0	1	0	1	0	1	0	0	0	1	1	0	1	0	0	1	1
MG error 2	0	0	0	0	0	0	0	0	0	0	0	0	0	1	0	1	0	1	0	1
MG error 3	0	1	0	0	0	0	1	0	1	0	0	0	0	0	0	1	1	0	0	0

NT/1.65 SD	40	42	44	47	49	51	55	57	59	62	66	67	70	74	75	78	79	80	81	85	95
CM rct 1	0	0	0	0	0	0	0	0	0	0	1	0	0	0	0	0	0	1	0	0	0
CM rct 2	0	0	0	0	0	0	0	0	0	0	1	0	0	0	0	0	0	0	0	0	1
CM rct 3	0	0	0	0	0	0	1	0	0	0	0	0	0	0	0	0	0	0	0	0	0
CM rct 4	0	0	0	0	0	0	1	0	0	0	0	0	0	0	0	0	0	0	0	0	1
CM rct 5	0	0	0	0	0	0	1	0	0	0	0	0	0	0	0	0	0	0	0	0	0
CM rct 6	0	0	0	0	0	0	1	0	0	0	1	0	0	0	0	0	0	0	0	0	0
CM error 1	0	0	0	0	0	0	0	0	0	0	1	0	0	0	0	0	0	0	0	0	1
CM error 2	0	0	0	0	0	0	0	0	0	0	1	0	0	0	0	0	0	0	0	0	1
CM error 3	0	0	0	0	0	0	0	0	0	0	1	0	0	0	0	0	0	0	0	0	1
CM error 4	0	0	0	0	0	0	0	0	0	0	1	0	0	0	0	0	0	0	0	0	1
CM error 5	0	0	0	0	0	0	0	0	0	0	1	0	0	0	0	0	0	0	0	0	1
CM error 6	0	0	0	0	0	0	0	0	0	0	1	0	0	0	0	0	0	0	0	0	1
MG rct 1	0	0	0	0	0	0	0	0	0	0	0	0	0	1	0	0	1	0	0	0	0
MG rct 2	0	0	0	0	0	0	0	0	0	0	0	0	0	1	0	0	1	0	0	0	0
MG rct 3	0	0	0	0	0	0	0	0	0	0	0	0	0	1	0	0	0	0	0	0	0
MG error 1	0	0	0	0	0	0	0	0	0	0	0	0	0	0	1	0	0	0	0	0	0
MG error 2	0	0	0	0	0	0	0	0	0	0	0	0	1	0	0	0	0	0	0	0	0
MG error 3	0	0	0	0	0	0	0	0	0	0	0	0	0	0	0	1	0	0	0	0	0

DYSL/1.65 SD	37	38	39	43	46	52	58	63	64	65	71	76	86	98	100	101	114	117	118	119
CM rct 1	0	0	1	0	0	0	0	0	0	0	0	0	0	0	0	0	0	0	0	0
CM rct 2	0	0	0	0	0	0	0	0	0	0	0	0	0	0	0	0	0	0	0	0
CM rct 3	0	0	0	0	0	0	0	0	0	0	0	0	0	0	0	0	0	0	0	0
CM rct 4	0	0	0	0	0	0	0	0	0	0	0	0	0	0	0	0	0	1	0	0
CM rct 5	0	0	0	0	0	0	0	0	0	0	0	0	0	0	0	0	0	0	0	0
CM rct 6	0	0	0	0	0	0	0	0	0	0	0	0	0	0	0	0	0	0	0	0
CM error 1	0	0	0	0	1	0	0	0	0	0	0	0	0	0	0	0	0	0	0	0
CM error 2	0	0	0	0	1	0	0	0	0	0	0	0	0	0	0	0	0	0	0	0
CM error 3	0	0	0	0	1	0	0	0	0	0	0	0	0	0	0	0	0	0	0	0
CM error 4	0	0	0	0	1	0	0	0	0	0	0	0	0	0	0	0	0	0	0	0
CM error 5	0	0	0	0	1	0	0	0	0	0	0	0	0	0	0	0	0	0	0	0
CM error 6	0	0	0	0	1	0	0	0	0	0	0	0	0	0	0	0	0	0	0	0
MG rct 1	0	0	0	0	0	0	0	0	0	0	0	0	0	0	0	0	0	0	0	0
MG rct 2	0	0	0	0	0	0	0	0	0	0	0	0	0	0	0	0	0	0	0	0
MG rct 3	0	0	0	0	0	0	0	0	0	0	0	0	0	0	0	0	0	0	0	0
MG error 1	0	0	0	0	1	0	0	0	0	0	0	0	0	1	0	1	0	0	0	0
MG error 2	0	0	0	0	0	0	0	0	0	0	0	0	0	0	0	0	0	0	0	1
MG error 3	0	0	0	0	0	0	1	0	0	0	0	0	0	0	0	1	1	0	0	0

Figure S9: deviating individual performance / visual (1SD and 1.65 SD)/ reaction time and errors of coherent motion and moving gratings depicted for different velocities; NT: neurotypical normal reading children, DYSL: children with dyslexia, numbers in the head row depict the individual subject, rct: reaction time, err: errors, CM: Coherent Motion, MG: Moving Gratings

NT/1.65 SD	40	42	44	47	49	51	55	57	59	62	66	67	70	74	75	78	79	80	81	85	95
tapping	1	0	0	0	0	0	0	0	0	0	0	0	0	0	0	0	0	1	0	0	0
statue	0	0	0	0	0	1	0	0	0	0	0	0	0	0	0	0	0	0	0	0	0

DYSL/1.65 SD	37	38	39	43	46	52	58	63	64	65	71	76	86	98	100	101	114	117	118	119
tapping	1	1	0	0	1	0	1	0	0	0	0	0	0	0	0	0	0	0	0	0
statue	0	0	0	0	0	0	0	0	0	0	1	0	0	0	0	0	0	0	0	1

Figure S10: deviating individual performance/cerebellar / subtestlevel (1.65 SD); NT: neurotypical normal reading children, DYSL: children with dyslexia, numbers in the head row depict the individual subject
